# Throat-Ail

**Published:** 1858-04

**Authors:** 


### HALL'S JOURNAL OF HEALTH.

OUR LEGITIMATE SCOPE IS ALMOST BOUNDLESS: FOR WHATEVER BEGETS PLEASURABLE AND HARMLESS FEELINGS, PROMOTES HEALTH; AND WHATEVER INDUCES DISAGREEABLE SENSATIONS, ENGENDERS DISEASE.

*We aim to show how Disease may be avoided, and that it is best, when sickness comes, to take no Medicine without consulting an educated Physician.*

VOL. VI.] APRIL, 1858. [No. 4.

### THROAT-AIL.

WHAT burly, straight-forward, direct, free-spoken, old fellows they were, who invented the English language! They loved a short, plain, unequivocal "yes" or "no;" delighted in monosyllables; hated circumlocution. And what a pity it is their degenerate descendants have fallen into such wordiness, that any one of them can talk an hour and say nothing; may write a book, and

"Leave not a trace behind,"

of one single idea! The two monosyllables above, how expressive! That wise-looking old gentleman yonder, with spectacles astride his nose, and the end of that resting on a gold-headed cane, with owl-like intelligence, peers down an immense cavern, its entrance lined with "ivories," "snaggles," gold, platina, lead, gutta percha, or caoutchouc, and pronounces, with oracular dignity, *Chronic Laryngitis!* And what a charming effect that has upon the patient! It quiets him at once!—not that he has the most remote conception of what those tremendous big words mean; but the Doctor has given a name, and if he can tell its name, "in course" he must know all about it. Thus the masses reason. But suppose the oracle had laid aside the "specs," had left his gold-headed cane at home, and, after close inquiry, and looking into the opened mouth, should have said, "Something is the matter with your throat," that same old gentleman would have been just nobody at all, in the patient's estimation; and the next minute, that patient would be in the street, muttering, in the deepest disappointment: "*Why, you good-for-nothing old Granny, I knew that before!*" Such is man in the mass.

"Throat-Ail!"—It carries the mind direct to the spot, localizes the disease, and gives it an appropriate designation—one which the most unintelligent can understand. But the effects of this "Throat-Ail" are so extraordinarily diverse, that many and many a time has the young doctor, in densest mazes of perplexity, exclaimed: "Oh, that there was more mathematics in medicine!" Equal to the despair of a very eminent Doctor of Divinity in this city, when we were children at school together. He "did our sums," as he loved mathematics; and we, in return, "parsed" his grammar lessons to him. In one of the first, we were trying to explain that a certain verb agreed with its nominative "*understood.*" He looked at us in blank bewilderment, as if to say, "Why not express the quantity? a thousand things might be understood, without its being the right understanding;" and he went off with a scowl on his face, and growling out "understood!" Thirty years later, we were listening to him in a large city. The same matter-of-fact, mathematical character of mind still distinguished him; not the slightest imagination whatever. It would take a year for him to write a verse of decent poetry; and as for attempting a flight of fancy, and playing the orator, it is an impossibility, as the reader will see. He was describing a visit to the Mammoth Cave in Kentucky; we saw him, in imagination, crossing the bottomless river in a frail canoe, in worse than midnight darkness, which the feeble torches only made the more distinct. We could appreciate it well, for we had been there ourselves, and we sympathized with the speaker, as up to that point, our experiences were alike; but further on, they were not: "For," said he, rising on his toes, and making a promiscuous gesture, "The boat turned over, the candle went out, and there we were!" and we have no doubt of the literal truth of the statement; for how could he have been anywhere else, under the circumstances, than "there?"

This same gentleman would always stick to the literal truth. He believed in mathematical fact, above all things. His father was as great in mind as his son now is, and headed the "Bar"

of his native State. "I will give you fifteen hundred dollars," said an eminent lawyer one day to Judge ——, "if you will find a 'fatal' flaw in this paper," handing him a slip with half a dozen lines; scarcely had he read it over, when he pointed with his finger, "there it is," and the money was paid over. But Judge —— could sooner ferret out a flaw in a written document, than in the character of his truthful son, who at the age of fourteen, perhaps, became a member of the Church. There was a regular prayer-meeting every week, which the minister always attended, and was accustomed to "draw out" the young men, by inviting them "to pray"—which, we have no doubt, the "divine" that was to be, did in secret; but to do so before the whole congregation, was a "sore trial."

The injunction to "attend the prayer-meeting" was imperative, and a prayer now and then was a certain consequence: the son was *in a strait betwixt two;* but he had 'mind' enough to extricate himself; he obeyed his father, attended the prayer-meeting as regularly as the day came; but without "leading in prayer." It was accomplished in this wise: He made it a point to get there before the minister, to notice all who came in, and selecting a pew, the Sunday occupants of which were pretty sure not to be there, he laid himself at full length on the seat, as soon as the minister came in, listened to every hymn given out, could rehearse the lecture, and give the names of every one who "joined in the exercises." The Judge was seldom present, being at court, but now and then he would chance to be in.

"My son, were you at the prayer-meeting this afternoon?"

"*Yes, Daddy.*"

"I did not see you."

"*But I was there.*"

As to the "subject treated," who prayed? "what hymns were sung?" every question was answered with mathematical accuracy.

With an inward resolve to watch more closely next time, and to wipe his spectacles with greater care, the worthy Judge changed the subject. But he died without the explanation. So much for Mathematics and Throat-Ail!

We were speaking of the varied effects of Throat-Ail on persons who complained of it. For example, the Rev. Mr. Smith is a country clergyman, the pastor or rector of a small congregation or parish. His salary is limited, barely adequate to his necessities, even with painful economies. He is busy all the time. What with funerals, preparation for the Sabbath, visiting the sick, sustaining the weak, cheering the despondent, waking up the freezing from their deathly sleep, startling the "hypocrite in Zion," and shaking the manacles of the lost over the worldly-minded, the fashionable, and the gay, he has not the means or facilities for taking care of himself as he should; and, before he knows it, he has taken cold, it settles in his throat, and a "tickling cough" or troublesome "hemming" remind him of some wrong going on somewhere; but he has no time for rest or medication, and works on. Hoarseness comes. It is an effort to speak, if not a decided pain. Despondency shows itself. He works in agony, and at last "gives up." And, as unreluctantly, sometimes, as if he were an old dray-horse, he is "turned out to grass," and—to die.

This is *Throat-Ail* from exposure and over-work.

But there is another "Brother," the revered Pastor of a rich city church, with his "five thousand a year." He works hard, and his people know it. They are very tender of him. No fond mother sees more quickly a "symptom" in her darling babe, than the members of the church descry some hidden meaning in the slightest "hack" or "hem;" as for an actual "cough," it wakes up everybody. "Something must be done;" and anon a "committee" of safety waits upon the minister, with a copy of some "resolutions," and a purse of some "acts," "to the end that" he spend a "vacation abroad." Well, the money is spent, and the vacation is spent, and the loved minister gladdens all eyes by ascending his pulpit as "hearty as a buck," and looking no more like "giving up just so," than the celebrated individual who first sat to the picture of that phrase.

Why *Throat-Ail* should send one man to the grave and another to Europe; one to a death in penury and inactivity, and another to "newness of life," may seem perplexing; but of the fact, however, there can be no doubt.

But not more "diverse" are the *effects* of Throat-Ail in these two cases, than were the "causes." In the former, the cause was exposure, and over-work; in the latter, the want of exposure, and there was not work enough. The country "Brother"

had not enough to eat (suitable): his fraternal city co-laborer had too much. The former worked too hard, for he had not time to rest: the latter worked too little, for the amount he ate. A cold, in the former case, led on to death: dyspepsia, in the latter, fired the train which would have ended as fatally, had not rest and recreation intervened to ward off the result.

But only think of attempting to cure both these ailments in the same way! by stuffing a swab of nitrate of silver down both throats; or at another "shop" filling the lungs with the *fumes* of liquor, and the stomach with the *fact*, the liquor itself.

In one case, the disease is in the stomach; in the other, in the lungs. Neither the liquor nor the "nitrate" ever cured anybody of either—the doctors of two hemispheres to the contrary, notwithstanding.

If the Throat-Ail arises from dyspepsia, then the stomach must be rectified: if it is caused by a cold, the lungs must be attended to. If the lungs are clearly at fault, then official rest and horseback exercise must be remedies: if the stomach does not perform its duty, then a journey abroad is appropriate; and when, in the latter case, the minister comes back greatly improved or entirely well, all are loud in their praises of a "European tour," and for a while everybody takes or recommends the same remedy, without respect to the cause, to the seat of the ailment, or to the patient's pecuniary ability to carry out the prescription. Hence, in many instances, the thoughtless advice is not only foolish, but heartless. But it is frequently observed, that before long, the returned minister has Throat-Ail again. Why? He comes back to the same system of disproportioned physical out-door exercise and eating: and the causes recurring, the effects very naturally reappear.

Therefore, to those who apply to us for advice, we often have occasion to say, "*Get well under the circumstances under which you expect to remain, and the same means which make you well, will keep you so.*" These remarks have been suggested by a letter received from an old patient, about two hours ago; they would have been more connected, had not our office been the play-ground of six children, from three to ten, not all our own quite; but they have this moment left—(wonder if we will write any better!)

Our patient had applied to us. Having the advantage of a rich father, he resigned his pastorate, because "everybody," that very sagacious intangibility, "advised it"—*then* came for our advice, in addition to "everybody's." "Advice" was good; and as his father was able to foot the bills, it was well enough to have plenty of it. We did not agree with "everybody," but gave our opinion, and he left. He did not "get on" fast enough; his wife became uneasy, and "everybody" was consulted again, resulting in the Medo-Persian edict, "Go South!"—and to the South he went. Fine thing to have plenty of money to go anywhere! What elephants he saw in the Florida everglades, and how many; what mosquitos and gallinippers he felt, and how large; what "bacon," and bacon only, he ate, and how hard, and black, and old; what cheerless hotels he "put up" at, destitute of chimneys, plaster, and pane of glass; what music he heard for the live-long night—of "keys" how varied, from the cough of a "beginner," to the cavernous ring of the poor skeleton of humanity, who was to see home, or mother, or wife, no more; what new sights he saw at every street-corner, of consumptives lean, long-faced, and bent, just tottering on the crumbling brink of the grave; how, believing in the virtues of air and exercise, he cut cord-wood six hours a day for months, "for nothing," with the thermometer at our summer heats; how he took it into his head that he ate too much, then lived on bread and water, working all the while; how he got no better, fast, rode a thousand miles on horseback, and reached New York worse than when he left it; how he came to our office, after a seven months' absence and silence—acknowledging that he knew less about himself and his constitution than he ever did before, and with what "*vim*" of gesture he declared he believed he was a fool; and how unanimously we agreed with him, *nem. con.*—we need not detail. We, however, encouraged him—to give us a check on his rich father, to begin at the beginning, and try it all over. Being "*fully persuaded in his own mind*," that he knew nothing about the physiological man, or medicine, he received our teachings with commendable humility. Without boring our readers with a long rigmarole as to what we did, and why, we, in general terms, give the "sum and substance" of our advice; it was to the effect, that as long as his heart was set on preaching, the more he didn't preach, the more he would not get well; that he ought to hunt up a place forthwith, or "take up" with the first offer made; to preach every day, and Sunday too, and leave the result to common sense.

As something or other would have it, of all the bleak, freezy, windy, piercingly cold situations in the Union, he was called to about the worst. He inquired of us what he should do? we counselled him to accept, pull off his coat, roll up his sleeves, and go to work; and that, too, the first winter after his Florida campaign! Let his very valuable and suggestive letter, dated Feb. 19, 1858, tell the remainder of the story, while we go to dinner, and see that the children have their "bibs" on, their food cut up in very small pieces before the "madam" makes her appearance as inspector-general, to see if all things have been properly done; for if not, who can describe—the remainder!

What a delightful thing it is to have everything to do, and more too, introducing us practically into that "variety" which *Cowper* affirms is the "*spice of life*," talking, singing, whistling, musing, tasting cheese, smelling butter, splitting wood for the cook, cleaning the side-walk for the housemaid, sleighing the children around the backyard, reading letters, *writing* for the "JOURNAL" whole pages of "common sense," *acting* for ourselves whole years of folly, exemplifying the wisdom of our writings by the practical exhibition of their opposites—believing, as we do, that contrasts are more striking than consistencies. Does anybody believe that the editor of this Journal avoids eating hot biscuits for supper because other people say they are unhealthy? or, that for the sake of "air and exercise," he commits the daily penance of a pilgrimage to some pillar, post, or tree, with the express design, when getting there, to turn round and trudge back again? Very far from it; we had rather romp with the children, split wood for the cook, and clear the side-walk of snow. Or, can it be supposed, that we are going to "harden" ourselves by dancing under a shower-bath of a winter's morning, or in summer either—then scrubbing the skin as if it were a grease-spot, with crash towels, horse-hair gloves, and steel files?—very clear of any such nonsense! We eat in moderation what is palatable to us, and have no "surplus" to scrub off. Nature cleans her own machinery, if let alone; or, if by accident, it "becomes foul," and she needs a little aid, the best scrubbing we know in practice is the human hand, the best lavement is delicious warm water. That is our practice, winter and summer; in both equally agreeable for face and hands, and entire person—always soothing, always safe. The results are, we have neither dyspepsia, gout, nor glooms. Glad to see anybody; practice daily the instinctive, wide-mouthed laugh, which rings into the street, and from cellar to attic. We seldom take cold, and sleep soundly from nine until daylight, winter and summer, and have, through it all, a heart and head literally and practically as soft as—dough!

Carrying our "contrasts" farther, we, full many a time and oft, come home with pockets stowed with cakes, candies, and toys for Alice and Bob; to whom, on either knee, before our office fire, as regularly as night-fall comes, shutters closed, the winter winds whistling in the darkness, with the bright gas-light within, we tell the "stories" of what we have seen or heard, or read, "once upon a time," &c. When their bed-time comes, we tarry awhile to fix on a button, or stop a stocking-hole, by sewing it up with brown thread—for then it stays stopped, according to our experience and observation, a great deal longer than if "darned" with yarn; for if yarn did not keep the hole away in the first place, we can't see how it would do it in the second. Perhaps our domestic reasoning is not as scientific as our professional; but we state our acts and deeds for information, and there leave them. At all events, this multitudinous diversity of engagements, with others not necessary to mention, being stated more at length in the "Life of the Caudles," to which the reader is respectfully referred—we repeat it—these diversities keep the mind from vegetating, and the blood from stagnation; they create a fine appetite, procure sound sleep, and a good time generally, from which that unfortunate class are for ever debarred, who have plenty of money and nothing to do; interested in nobody, nobody interested in them. Be assured, ye poverty-stricken, that the necessity of a vigilant activity is a happier inheritance than that of piles of glittering, heart-hardening gold.

These things being so, it is easy to see, that between them and "Throat-Ail" there is no connection whatever; therefore, we hasten to give the very valuable, because practical, truthful, and judicious letter of our former patient.

"My Dear Doctor—I notice, in the last number of your Journal, an invitation to your old patients to respond to the question 'How are you?' When I first read it, I inwardly resolved to write and let you know, at the first convenient opportunity. Lectures, sermons, literary addresses, parochial visiting, and a pile of other engagements, have prevented till now. In the first place, let me say, that I always read your Journal through as soon as I take it from the office. Other engagements always give way to that. Besides that, I intersperse divinity and sermonizing, with an hour a day on physiology and hygiene Have found this sort of literature useful. You know my infirmity—a lazy liver. How to manage with it has sometimes been almost as perplexed a question as for the sailors to keep the ship from foundering with Jonah under the hatches. I have felt my way to a variety of devices. First of all, the fable of the hare and the tortoise has been of use. Stomach=hare; liver=tortoise—now for the race. What an unequal strife! A jockey or a glutton would bet ten to one on the stomach; but if I can get the nimble fellow asleep by skipping a meal, and, at the same time, keep the liver jogging on by a brisk walk, or ride in the saddle, I find I can often bring the poor stupid thing through in time. This seems to me only a practical sequence from your direction to eat nothing after the time for a "move" has passed, till the bowels do their duty.

"Again, I get up betimes in the morning at four o'clock, thus waking up my liver, and getting it on the road two hours before the stomach is harnessed up by breakfast. This is another of your priceless precepts.

"Again, I have been studying dietetics, theoretically and practically; and find that I can do much in this way. I often live for days on bread and apple-sauce, cutting the acquaintance of beef and mutton. I feel not the slightest diminution of strength by so doing, and it aids in cooling the blood, and carrying away surplus bile, besides taking some of the saffron from my cheeks. Moreover, I have found that my mind works more freely on a very little meat, than when I make it the central article of the table. Well, you begin to apprehend that I shall join the vegetarian society? Not quite yet; but you will indorse the opinion that meat in great proportion clogs the intellect, will you not?

"All the above seems rather to smack of egotism, rather like the portly, big-bellied Englishman's 'I, myself.' But you invited your cast-off patients to talk about themselves, and here is a specimen. You know I was troubled with a torrent of mucus in the form of expectoration. I have reduced it to a pretty normal proportion by temperance and fresh air. But when the Winter winds began to blow last Fall, I had discharged such an amount of carbon, that I was ready to freeze to death, and had to pile on flannels by day, and blankets by night. Have got warmer now, however; and this morning rode on horseback about eight miles, mercury about zero, with plenty of caloric 'from the crown of the foot to the toe of the heel,' as you said once.

"The people here say that —— is remarkably exempt from consumption of the lungs. I am not so sure of that. I have already buried two of my congregation who died of it, and have met with many another with sore throat, bronchitis, and kindred ailments. For myself, I would prefer a *drier* air on the whole, with fewer high winds; nevertheless, have not had the faintest symptom of cough or sore throat since I came here, and only one cold in six months, and that the result of sheer carelessness, but frightened it away in three days with bread and water. My family are all well, children fat, rosy-cheeked, and hearty. Generally, my spirits are good; if I find myself getting fretful, am pretty sure that I have overtaxed the stomach, and just cut short the rations, to get elastic again, and ready for a laugh. Need I say, I owe much to you? I should be ungrateful to forget it, or not to speak of it. I should be glad to hear from you at any time; and if you think I can write anything that will be useful in your Journal, do let me know what to write about, and most gladly will I do it. If I could give to all literary dyspeptics an account of the trotting-match between my nimble stomach and my lazy liver, I should like to do it; perhaps it would suggest a useful idea to some of them.

"Sincerely yours."

